# Protective effects of flavonol isoquercitrin, against 6-hydroxy dopamine (6-OHDA) - induced toxicity in PC12 cells

**DOI:** 10.1186/1756-0500-7-49

**Published:** 2014-01-21

**Authors:** Kasthuri Bai Magalingam, Ammu Radhakrishnan, Nagaraja Haleagrahara

**Affiliations:** 1 Department of Pathology, Faculty of Medicine, International Medical University, Kuala Lumpur, Malaysia; 2 Discipline of Physiology and Pharmacology, School of Veterinary and Biomedical Sciences, Faculty of Medicine, Health and Molecular Sciences, James Cook University, Townsville 4811, Australia

**Keywords:** Antioxidant Flavonoids 6-hydroxydopamine Parkinson’s disease Oxidative stress

## Abstract

**Background:**

Free radicals-induced neurodegeneration is one of the many causes of Parkinson’s disease (PD). This study investigated the neuroprotective effects of flavonol isoquercitrin against toxicity induced by 6-hydroxy-dopamine (6-OHDA) in rat pheochromocytoma (PC12) cells.

**Methods:**

PC12 cells were pretreated with different concentrations of isoquercitrin for 4, 8 and 12 hours and incubated with 6-OHDA for 24 hours to induce oxidative cell damage.

**Results:**

A significant cytoprotective activity was observed in isoquercitrin pre-treated cells in a dose-dependent manner. There was a significant increase (P < 0.01) in the antioxidant enzymes namely superoxide dismutase, catalase, glutathione peroxidase, and glutathione in isoquercitrin pretreated cells compared to cells incubated with 6-OHDA alone. Isoquercitrin significantly reduced (P < 0.01) lipid peroxidation in 6-OHDA treated cells. These results suggested that isoquercitrin protects PC 12 cells against 6-OHDA–induced oxidative stress.

**Conclusions:**

The present study suggests the protective role of isoquercitrin on 6-hydroxydopamine-induced toxicity by virtue of its antioxidant potential. Isoquercitrin could be a potential therapeutic agent against neurodegeneration in Parkinson’s disease.

## Background

Parkinson’s disease (PD) is a neurodegenerative disease involving the degeneration of dopaminergic neurons in the striatum. The selective loss of dopaminergic neurons in the substantia nigra is the primary neuropathology in PD [1-3]. One of the many causes of PD is the accumulation of free radicals and oxidative stress which leads to the selective neuronal loss [4-6]. Though great advances have been made in the development of novel drugs to treat this disease, the appropriate pharmacological agent for PD is still elusive [3,7]. Alternative therapy in PD is aimed to effectively prevent the progression of neurodegeneration process hence improving the clinical presentation of PD including tremor, bradykinesia, rigor and hypokinesia [8]. Free radical scavengers such as antioxidant agents may be helpful in prolonging the survival of dopaminergic neurons. Flavonoids are naturally occurring polyphenol compounds widely distributed throughout the plant kingdom [9,10]. They exhibit a variety of biological activities, such as anti-oxidation, anti-inflammation, anti-bacteria, and anti-allergy [11-14]. Many of these flavonoids act as neuroprotective agents in many neurological disorders [4,15].

Isoquercitrin, also known as quercetin 3-glucoside, is a glucose-bound derivative of quercetin, and is reported to have anti-inflammatory and antioxidant activities [5,6]. Isoquercitrin is widely found in mangoes, rheum nobile, apples, onions and in many other fruits and vegetables [16,17]. It is considered to be a “bio-quercetin” without the potential adverse effect of quercetin [18]. Although several studies have demonstrated the antioxidant property of isoquercitrin [5,6,16,17], but the neuroprotective potential of isoquercitrin is not well explored. Hence, the objectives of our study are to establish the neuroprotective role of isoquercitrin as well as to elucidate the antioxidant mechanisms of isoquercitrin in 6-OHDA-induced neurotoxicity in PC12 neuronal cells. PC12 cells are commonly used in the investigation of neurotherapeutics study for Parkinson’s disease. These cells are known to secrete dopamine neurotransmitter and contain high amounts of dopamine transporters. The cell line is derived from the rat pheochromocytoma and is frequently used as an in vitro model to study neuronal toxicities of drugs on central dopaminergic neurons [19,20].

## Results

### Dose response of 6-OHDA toxicity

**Figure 1:**
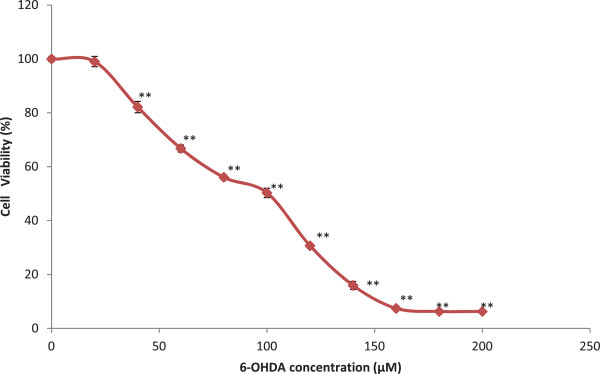
**The dose–response of different concentrations of 6-hydroxydopamine (6-OHDA) (0–200 μM) used to induce neurodegeneration in 1 × 10**^**5 **^**cells/well.** Pheochromocytoma (PC 12) cells for the duration of 24 h. The viability of PC12 cells was determined using the MTT reduction assay. Values are the percentages of viable cells, with the viability of untreated control cells taken as 100%. Data are mean and S.E. values from three independent experiments (n = 4). **p < 0.01, relative to untreated cells.

The effect of different concentrations of 6-OHDA was assessed on 10,000 cells/well to determine the concentration of 6-OHDA which results in 50% of cell inhibition (IC_50_ value). The results showed a significant decline of cell viability following 24 hours of incubation of PC12 cells with an increasing concentration of 6-OHDA (0 – 200 μM). The 6-OHDA concentration which resulted in 50% PC12 cell inhibition was 100 μM (50.33 ± 1.72) compared to the negative control group. The mean percentage of cell viability of each group was compared with the mean percentage of untreated control and reported as mean ± SEM (Figure 1).

### Evidence of isoquercitrin in protecting 6-OHDA- induced oxidative stress

**Figure 2:**
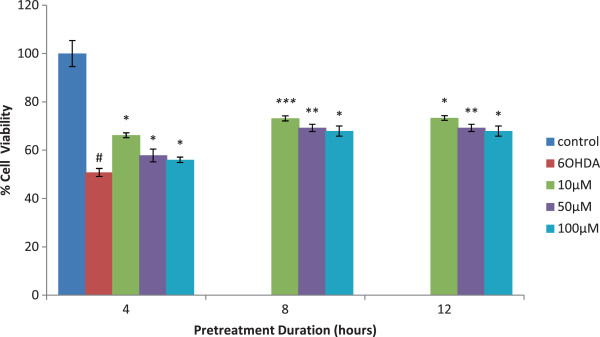
**Assessment of cell viability after pretreatment with isoquercitrin.** The isoquercitrin pre - treatment showed a significant increase in cell viability compared to cells treated with 6-OHDA alone. The cell viability was the highest at 10 μM of isoquercitrin at all the three durations of time. Cell viability increased as the cells exposed for 12 hrs. with isoquercitrin. Values are the percentages of viable cells, with the viability of untreated control cells (control) taken as 100%. Data are mean and S.E. values from three independent experiments (n = 3). #*p* < 0.05, relative to control cells. *p < 0.05, **p < 0.01 and ***p < 0.001, relative to cells treated only with 6-OHDA.

Isoquercitrin pre-treatment showed a significant increase in cell viability compared to cells treated with 6-OHDA alone. The 100 μM of 6-OHDA alone resulted in cell viability of 50.80 ± 1.66. The cell viability was the highest at 10 μM of isoquercitrin at all the three durations of time. However, the optimum reading was recorded at 10 μM of isoquercitrin at 8 hours of pretreatment (73.2 ± 1.01) with *p* < 0.001. The cell viability was also high as the cells exposed for longer durations with isoquercitrin which is at 12 hours (Figure 2). These findings suggest that isoquercitrin could activate the native antioxidant mechanisms in the PC12 cells during the pretreatment phase, which was able to protect these cells from undergoing neurodegeneration process.

### Glutathione

**Figure 3:**
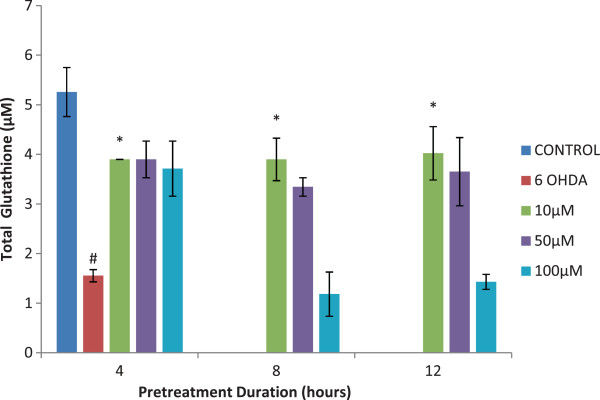
**Effect of isoquercitrin on glutathione level.** There are significantly higher glutathione in the control sample and significantly lower in samples incubated with 6-OHDA. The isoquercitrin pretreatment increased the glutathione level in 6-OHDA induced PC12 cells. Data are mean and S.E. values from three independent experiments (n = 3). #*p* < 0.05, relative to control cells. *p < 0.05, relative to cells treated only with 6-OHDA.

The glutathione level was significantly higher in the untreated sample and lower in samples incubated with 6-OHDA alone (p < 0.001). All samples incubated with isoquercitrin demonstrated higher amount of glutathione concentrations (p < 0.001). The glutathione concentration was indirectly proportional to the isoquercitrin concentrations. Isoquercitrin pretreatment at 10 μM, showed the highest glutathione concentration in all pretreatment durations (4, 8 and 12 hours) with p < 0.05 relative to 6-OHDA treated group (Figure 3). However, the level of total glutathione was very much decreased at 100 μM of isoquercitrin at 8 and 12 hour of pretreatment compared to the 4 hours of pretreatment.

### Glutathione peroxidase

**Figure 4:**
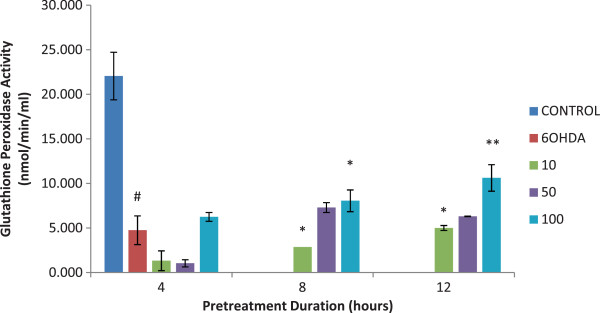
**Effect of isoquercitrin on glutathione peroxidase activity.** The enzyme level was significantly higher in isoquercitrin pretreated samples. Glutathione peroxidase activity increased in a dose dependent manner. Data are mean and S.E. values from three independent experiments (n = 3). #*p* < 0.05, relative to control cells. *p < 0.05 and **p < 0.01, relative to cells treated only with 6-OHDA.

The GPx enzyme level was significantly higher in isoquercitrin pretreated samples compared to the sample incubated with 6-OHDA alone (p < 0.001). The enzyme activity increased in a dose dependent manner and the highest activity was found at 100 μM of isoquercitrin pre-treated at 8 and 12 hours of incubation period. (*p* < 0.05 and *p* < 0.01). However, isoquercitrin did not stimulate the GPx activity at concentrations lower than 100 μM at all pretreatment periods as shown in Figure 4.

### Superoxide dismutase (SOD)

**Figure 5:**
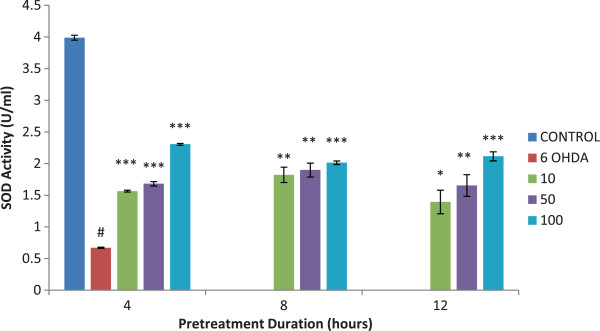
**Effect of isoquercitrin on superoxide dismutase activity.** The SOD significantly increased in all isoquercitrin pretreated cells. There was a significant elevation of SOD with the presence of isoquercitrin in a dose-dependent manner. Data are mean and S.E. values from three independent experiments (n = 3). #*p* < 0.001, relative to control cells. *p < 0.05, **p < 0.01 and ***p < 0.001, relative to cells treated only with 6-OHDA.

The superoxide dismutase level was significantly increased (p < 0.01) in all isoquercitrin pretreated PC12 cells. The SOD activity did not change to a greater level as the duration of exposure with isoquercitrin was increased. However, the assay showed statistically significant elevation of SOD activity with the presence of isoquercitrin in a dose-dependent manner (p < 0.01) (Figure 5).

### Catalase

**Figure 6:**
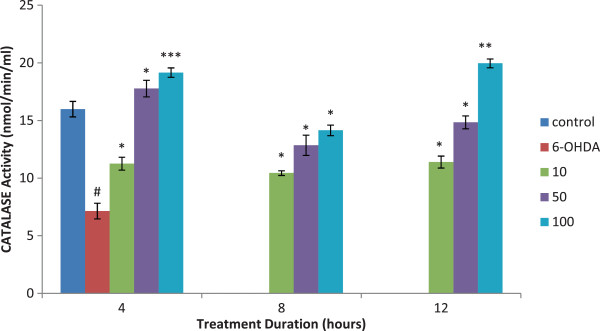
**Effect of isoquercitrin on catalase activity.** Isoquercitrin showed a significant increase in the catalase activity in all pretreated cells in a dose dependent pattern. The greater activity of catalase was seen after 4 and 12 hours. Data are mean and S.E. values from three independent experiments (n = 3). #*p* < 0.01, relative to control cells.*p < 0.05, **p < 0.01 and ***p < 0.001, relative to cells treated only with 6-OHDA.

Isoquercitrin showed a significant increase (p < 0.01) in catalase activity in all pretreated cells in a dose dependent pattern. Isoquercitrin pretreated PC12 cells showed a marked increase in catalase activity in a dose-dependent manner as shown in Figure 6. The highest catalase activity was observed at 100 μM of isoquercitrin at 4, 8 and 12 hours of pretreatments. Therefore, we can suggest that catalase enzymes was greatly suppressed during oxidative stress (6-OHDA alone) and markedly expressed in the presence of isoquercitrin (Figure 6).

### Lipid peroxidation

**Figure 7:**
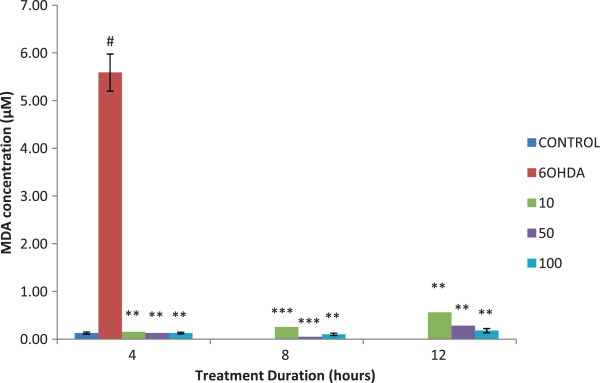
**Effect of isoquercitrin on lipid peroxidation.** Isoquercitrin significantly reduced the lipid peroxidation in a dose-dependent manner. The highest concentration of isoquercitrin showed the greatest potential in suppressing lipid peroxidation in PC12 cells. Data are mean and S.E. values from three independent experiments (n = 3). #*p* < 0.01, relative to control cells. **p < 0.01 and ***p < 0.001, relative to cells treated only with 6-OHDA.

A significant reduction of malondialdehyde level was observed in cells treated with isoquercitrin. PC12 cells treated with 6-OHDA alone showed a remarkably high level of malondialdehyde which is an indication of lipid peroxidation or oxidative stress. Isoquercitrin significantly reduced the lipid peroxidation in a dose-dependent manner. The highest concentration of isoquercitrin showed the greatest potential in suppressing lipid peroxidation in PC12 cells as the MDA level was the least in that group (100 μM). All the test groups displayed significant results with p < 0.01 relative to 6-OHDA treated cells alone (Figure 7).

## Discussion

Many lines of evidence have proven the oxidative stress due to imbalance in the free radical generation and endogenous antioxidant defense system may lead to the selective neuronal loss in Parkinson’s disease [21-23]. Hence, flavonoid polyphenols, particularly isoquercitrin can be an ideal candidate as neuroprotective agent to cease or delay the degeneration of dopaminergic cells [24,25]. In our study, the neuroprotective role of isoquercitrin, a flavonoid glycoside was investigated using a 6-OHDA induced PC12 cellular model of Parkinson’s disease. In the cell viability assay, isoquercitrin pretreatment has demonstrated a remarkable increase of cell viability at 12 hours of incubation with 10 μM of isoquercitrin. The isoquercitrin pretreated prior to 6-OHDA exposure resulted in stimulation of antioxidant enzyme activity in the neuronal cells as the cells are more resilient in coping with upcoming oxidative stress [26,27]. The antioxidant enzyme defense system functions in eliminating the free radical induced cellular damage during the defense against microorganisms, toxic chemicals and other conditions of cellular stress [20,21,28].

To further validate the neuroprotective role of isoquercitrin, the antioxidant enzyme status of isoquercitrin pretreated PC12 cells was assessed to confirm the antioxidant capability of this flavonol. The antioxidant enzymes which were studied are superoxide dismutase (SOD), catalase (CAT), glutathione and glutathione peroxidase (GPx). SOD occur in significantly higher amounts in the brain and these enzymes, which are of three types (Cu-Zn SOD, Mn-SOD and EC-SOD) readily catalyze the dismutation of the superoxide anion to oxygen molecules and hydrogen peroxide, a less toxic molecule [21,29]. There was a significant increase in SOD in all the isoquercitrin pretreated cells in a dose dependent manner. This increase in SOD activity proved that there was a direct activation of SOD by isoquercitrin to catalyze the superoxide anions produced by 6-OHDA. Catalase, a tetrameric structure with four indistinguishable tetrahedrally arranged residues with a single ferri-protoporphyrin subunit is ubiquitously found in the liver, kidney and erythrocytes [30]. Catalase accounts for detoxifying H_2_O_2_ molecules, whereby it is converted to oxygen and water molecule and this reaction through a reaction known as the catalytic reaction [31]. The CAT activity in isoquercitrin pretreated 6-OHDA induced PC12 cells showed a statistically significant effect in all the treatment groups. The CAT activity in the 6-OHDA treated group was significantly reduced compared to control group. The increased CAT activity could be due to two mechanisms, (i) increased in hydrogen peroxide molecules by SOD triggered the CAT enzyme, (ii) isoquercitrin caused direct activation of CAT enzyme which catalyzed the toxic hydrogen peroxide to water and oxygen molecules [29,32].

Glutathione peroxidase, which is largely found in the cytoplasm and mitochondria of eukaryotic cells is a vital antioxidant enzyme that catalyzes the reduction of hydroperoxides [33]. In this study, the GPx/Glutathione activity was also increased by antioxidant treatment. Isoquercitrin probably interacted with GPx and glutathione to enhance their antioxidant activity in PC12 cells [21,34]. Malondialdehyde (MDA) is a naturally occurring product of lipid peroxidation that will react with the thiobarbituric acid (TBA) and form MDA-TBA adducts [35]. Malondialdehyde produced due to lipid peroxidation accumulated in the cells and cause cell damage. MDA increased in 6-OHDA treated cells and isoquercitrin effectively reduced the levels of MDA in the pre-treated cells [27-29,32].

Free radicals generated by neurotoxin, 6-hydroxydopamine caused neuronal cell loss via DNA defects, lipid peroxidation and cytoskeletal disorganization [34]. Moreover, studies also demonstrated that 6-OHDA induced neuronal loss was due to inhibition of the mitochondria respiratory chain complexes I and IV, oxidative phosphorylation uncoupling, mitochondrial membrane potential collapse [4,22,35,36]. In this study, we have proved that 6-hydoxydopamine induced oxidative stress and cell death by decreasing the scavenging enzymes (SOD, catalase and GPx) in PC 12 cells. Isoquercitrin pretreatment caused a significant elevation in these scavenging enzyme levels and attenuate oxidative damage to the cells [22,37,38]. Isoquercitrin helped to maintain the levels of these antioxidant enzymes and suppress lipid peroxidation as well as protect the neuronal cell from undergoing cell death.

## Conclusions

The results of this study confirmed that neurotoxin 6-hydroxy dopamine causes suppression of antioxidant enzyme levels in PC 12 cells, which could be a reason for the progressive neuronal death. Isoquercitrin’s role in protecting against 6-OHDA induced oxidative stress, suggests that this flavonol may serve as a potential neuroprotective agent against the underlying pathology associated with neurodegenerative diseases like Parkinson’s disease.

## Methods

### Materials

PC12 cells were purchased from ATCC (#CRL-1721.1 PC12 ADH, RattusNorvegicus). 6-hydroxydopamine, isoquercitrin, poly-L-Lysine, MTT (3-(4,5-Dimethylthiazol-2-yl)-2,5-diphenyltetrazolium bromide),3,4 – dihydroxy-L-phenylalanine (Levodopa) and dimethyl sulphoxide (DMSO) were purchased from Sigma-Aldrich (USA). Dulbecco’s modified eagle’s medium (DMEM), pen-strep, horse serum, and fetal bovine serum was purchased from GIBCO Inc. (USA). Kits for glutathione peroxidase, superoxide dismutase, catalase, thiobarbiturate assay, and glutathione were purchased from the Cayman Chemical Company (USA).

### Cell culture

PC12 cells were grown in a humidified incubator with 5% CO_2_ at a temperature of 37°C in DMEM medium supplemented with 5% horse serum and 5% fetal bovine serum and pen-step (100 U/ml). The cells were cultured in poly-L-lysine coated T-75 culture flasks. The cells used in the experiments were taken between passage 2 and 8 as cells tend to get clumpy and difficult to isolate after passage 10. When the cells were 80% confluent, they were dislodged from the flask using a cell scraper. The dispersed cells were grown on poly-L-lysine coated 96-well microplate at a density of 1 × 10^5^ cells/ml and allowed overnight incubation to facilitate cell adhesion to the substrate. The cells were treated for 4, 8 and 12 hours in the presence of isoquercitrin at three different concentrations, 10 μM, 50 μM and 100 μM. Subsequently, the pre-treated cells were induced using 6-OHDA for 24 hours and assayed for its antioxidant activities. Control cells were cultured in complete DMEM alone and positive control cells were treated only with 6-OHDA.

### Determination of cell viability

The cytotoxicity effect of isoquercitrin pretreatment on 6-OHDA induced PC12 was determined using MTT (3-(4,5-Dimethylthiazol-2-yl)-2,5-diphenyltetrazolium bromide assay. MTT, a yellow tetrazole, is reduced to insoluble purple formazan in the mitochondria of viable cells and appears purple. The insoluble purple formazan was dissolved using a solubilizing solvent and the colored solution was measured at 570 nm using a microplate-reader. Data on cell viability were expressed as percentage of the surviving control cell in the study.

### Biochemical parameters

Glutathione assay kit utilizes an optimized enzymatic recycling method that uses glutathione reductase for quantification of GSH. Rate of production of yellow colored 5-Thio-2-nitrobenzoic acid (TNB) is directly proportional to the recycling reaction and concentration of glutathione in the sample. Lipid peroxides, derived from polyunsaturated fatty acids, are unstable and decompose to form a complex series of compounds which include reactive carbonyl compounds, such as MDA. MDA-TBA adducts formed by the reaction of MDA and TBA under high temperature and acidic conditions is measured colorimetrically at 550 nm. Super oxide dismutase assay kit utilizes a tetrazolium salt for the detection of superoxide radicals (O2–) generated by xanthine oxidase and hypoxanthine. One unit of SOD is defined as the amount of an enzyme necessary to exhibit 50% dismutation of superoxide radical. Superoxide dismutase levels were determined from a standard curve and expressed as U/ml of protein. Glutathione peroxidase assay kit measures GPx activity indirectly by a coupled reaction with glutathione reductase. Oxidized glutathione, produced upon reduction of an organic hydroperoxide by GPx, is recycled to its reduced state by glutathione reductase and NADPH. The oxidation of NADPH to NADP + is accompanied by a decrease in absorbance at 340 nm. Glutathione peroxidase levels were determined from a standard curve and expressed as nmol/min/ml of protein. The Cayman chemical catalase kit utilizes the peroxidatic function of catalase for determination of enzyme activity. The assay is based on the reaction of catalase with methanol in the presence of an optimal concentration of H2O2. The formaldehyde produced is measured spectrophotometrically with 4-amino-3-hydrazino-5-mercapto-1,2,4-triazole as the chromogen.

### Statistical analysis

Data were expressed as mean ± SEM. The results were analyzed using analysis of variance (ANOVA) using SPSS Inc. software (SPSS Statistics, V20.0.0). Differences between treatment groups were determined using the Bonferonni post-hoc test. A value of P < 0.05 was considered statistically significant.

## Competing interests

The authors declare that they have no competing interests.

## Authors’ contributions

KBM performed all experiments and wrote the manuscript. AR and HN designed the overall study and prepared the final manuscript. All authors read and approved the final manuscript.
